# A Multicenter Retrospective Study of Epidemiological Trends in Benign Acute Childhood Myositis Before and After the COVID-19 Pandemic

**DOI:** 10.7759/cureus.91635

**Published:** 2025-09-04

**Authors:** Mattia Pasquinucci, Asia Calgaro, Massimo Scollo, Davide Meneghesso

**Affiliations:** 1 Department of Neuroscience, Rehabilitation, Ophthalmology, Genetics, and Maternal and Child Health, IRCCS (Istituto di Ricovero e Cura a Carattere Scientifico) Istituto Giannina Gaslini, Genoa, ITA; 2 Department of Pediatrics, AULSS 7 Pedemontana (Azienda Unità Locale Socio Sanitaria), San Bassiano Hospital, Bassano del Grappa, ITA; 3 Department of Pediatrics, Università degli Studi di Padova, Padua, ITA; 4 Department of Pediatrics, AULSS 7 Pedemontana (Azienda Unità Locale Socio Sanitaria), Alto Vicentino Hospital, Santorso, ITA

**Keywords:** benign acute childhood myositis, influenza virus, renal impairment, sars-cov-2, viral infection

## Abstract

Background

Benign acute childhood myositis (BACM) is a rare condition that usually follows viral infections, predominantly affecting school-aged males. At onset, patients typically complain of soreness in the lower limbs and bilateral calves, with gait abnormalities such as refusal to bear weight, a wide-based gait, or toe walking. This is a generally self-limiting condition, but it may require hospitalization for the initiation of intravenous hydration in severe cases. Renal involvement due to rhabdomyolysis is extremely rare. The diagnostic work-up is often limited by the lack of awareness among pediatricians. This study aimed to describe its epidemiological and clinical characteristics, summarizing cases before and after the COVID-19 pandemic, and providing suggestions for the management of this condition.

Methods

This retrospective multicenter study used data from the AULSS 7 Pedemontana database. We included consecutive patients with clinical and biochemical diagnosis of BACM who were admitted to the Emergency Departments of three Italian hospitals between January 1, 2012, and December 31, 2024. Patients were identified through an extensive search using discharge codes according to the International Classification of Diseases, 9th Revision, Clinical Modification. Statistical analyses were performed using IBM SPSS Statistics for Windows, Version 21 (Released 2013; IBM Corp., Armonk, New York, United States).

Results

A total of 55 episodes of childhood myositis affecting 51 patients were identified. Most patients exhibited fever, rhinitis, pharyngitis, asthenia, and hyporexia; a smaller proportion presented with gastrointestinal symptoms. Consistent with the current literature, the predominant symptom of myositis was lower limb pain (n = 49, 89%), followed by refusal to ambulate (n = 30, 55%), often characterized by a wide-based gait (n = 17, 31%), typically following febrile illness. The incidence apparently increased in the post-pandemic period, as did the mean creatine phosphokinase levels increase from admission (928 IU/L vs. 146 IU/L, p = 0.03). A slower normalization of creatine phosphokinase values was also observed during the post-pandemic period (8.5 days vs. 4.2 days, p = 0.004). A higher hospitalization rate was noted in the post-pandemic group (n = 21, 70%) compared to the pre-pandemic group (n = 12, 48%), although this difference did not reach statistical significance (p = 0.10). The need for hospitalization was directly associated with elevated creatine phosphokinase levels (p = 0.05), renal impairment (p = 0.05), elevated blood urea levels (p < 0.0001), older age (p = 0.04), and inversely correlated with the presence of gastrointestinal symptoms (p = 0.001).

Conclusion

While it remains benign, a standardized approach is needed for childhood myositis diagnosis and management, balancing the risks of unnecessary hospitalizations and potential complications. All patients should undergo blood tests along with a urine dipstick, electrocardiogram, and, if possible, influenza A/B swab testing. Patients with mild symptoms, low muscle enzyme levels, no renal impairment, and good family compliance may be discharged home with parental reassurance about the benign nature of the condition. Otherwise, we suggest hospitalization or short-term observation for intravenous hydration and laboratory monitoring. Further research is warranted to elucidate potential post-pandemic changes in disease presentation.

## Introduction

Benign acute childhood myositis (BACM) is a rare complication that can occur during systemic infections caused by certain pathogens, typically viral. BACM can present as either a sporadic or, occasionally, an epidemic condition [1]. The most affected individuals are generally preschool-aged male children. This demographic distribution has not yet been fully explained, and it is suspected that there may be a genetic predisposition to the development of this condition [2].

The microorganisms most frequently involved include influenza B virus, influenza A virus, parainfluenza virus, adenovirus, rotavirus, Epstein-Barr virus (EBV), cytomegalovirus (CMV), respiratory syncytial virus (RSV), and enterovirus (EV). Recently, human coronaviruses (hCoV) such as severe acute respiratory syndrome coronavirus 2 (SARS-CoV-2) have also been described as an emerging cause since the beginning of the pandemic. Among bacterial pathogens, cases have been described due to atypical bacteria such as *Mycoplasma pneumoniae* and Gram-positive bacteria like *Streptococcus pyogenes* [3]. BACM is characterized by a sudden onset of muscle pain and weakness. BACM can often lead to significant parental concern and misdiagnosis. Unlike more severe myopathies, BACM is generally self-limiting, with most children experiencing a full recovery within weeks. It generally manifests a few days after the onset of flu-like symptoms, with upper respiratory tract symptoms and fever. Gastrointestinal symptoms and skin rash are rarely reported. At onset, patients typically complain of soreness in the lower limbs and bilateral calves, with gait abnormalities such as refusal to bear weight, a wide-based gait, or toe walking. Physical examination may reveal tenderness upon palpation of the large muscle groups of the lower limbs and calves, in the absence of other significant neurological deficits or joint involvement [4]. Laboratory findings, in addition to elevated creatine phosphokinase (CPK), generally include increased C-reactive protein (CRP), lactate dehydrogenase (LDH), and transaminases, with concurrent virus-induced neutropenia. No abnormalities or alterations have been described in the smooth or cardiac muscle.

This is a generally self-limiting condition, but may require hospitalization for the initiation of intravenous hydration in severe cases [5]. Renal involvement due to rhabdomyolysis is an extremely rare but important complication to recognize and treat promptly [6]. The diagnostic work-up for BACM is often limited by the lack of awareness among pediatricians. There are often delays in diagnosis, frequently due to underestimation of the clinical picture and misdiagnosis with virus-related myalgias, and delays in treatment. The finding of elevated CPK levels can be particularly alarming for parents and, sometimes, for medical personnel [7].

Objectives

This study aims to describe the epidemiology and clinical characteristics of BACM cases presenting to pediatric emergency departments (EDs). We will compare the patient populations affected during the pre-pandemic period (2012-2019) and the post-pandemic period (2020-2024). A second aim is to provide suggestions for the management of this condition.

## Materials and methods

Study design

This study is a retrospective multicenter collection of consecutive patients with a clinical and biochemical diagnosis of BACM, conducted between January 1, 2012, and December 31, 2024. Patients were identified at the pediatric EDs of the hospitals in Bassano del Grappa, Santorso, and Asiago (Vicenza, Italy). Patients were identified through an extensive search using the following International Classification of Diseases, 9th Revision, Clinical Modification (ICD-9-CM) discharge codes: 728.0 (infective myositis), 718.81 (interstitial myositis), 728.88 (rhabdomyolysis), 728.89 (other disorders of muscle, ligament, and fascia), 728.9 (unspecified disorder of muscle, ligament, and fascia), 729.1 (myalgia and myositis), 359.89 (other myopathies), and 359.9 (myopathy). The diagnosis of BACM was defined as the presence of symptoms affecting the lower limbs (such as altered or refusal of walking, spontaneous calf pain, or pain on palpation of the muscle masses) associated with elevated CPK levels above the normal range (150 U/L) in the context of an acute infectious episode. Exclusion criteria included diagnoses of pyomyositis, dermatomyositis, polymyositis, known neuromuscular pathology, and myalgia during viral infection without an increase in CPK.

Sample size selection

**Figure 1:**
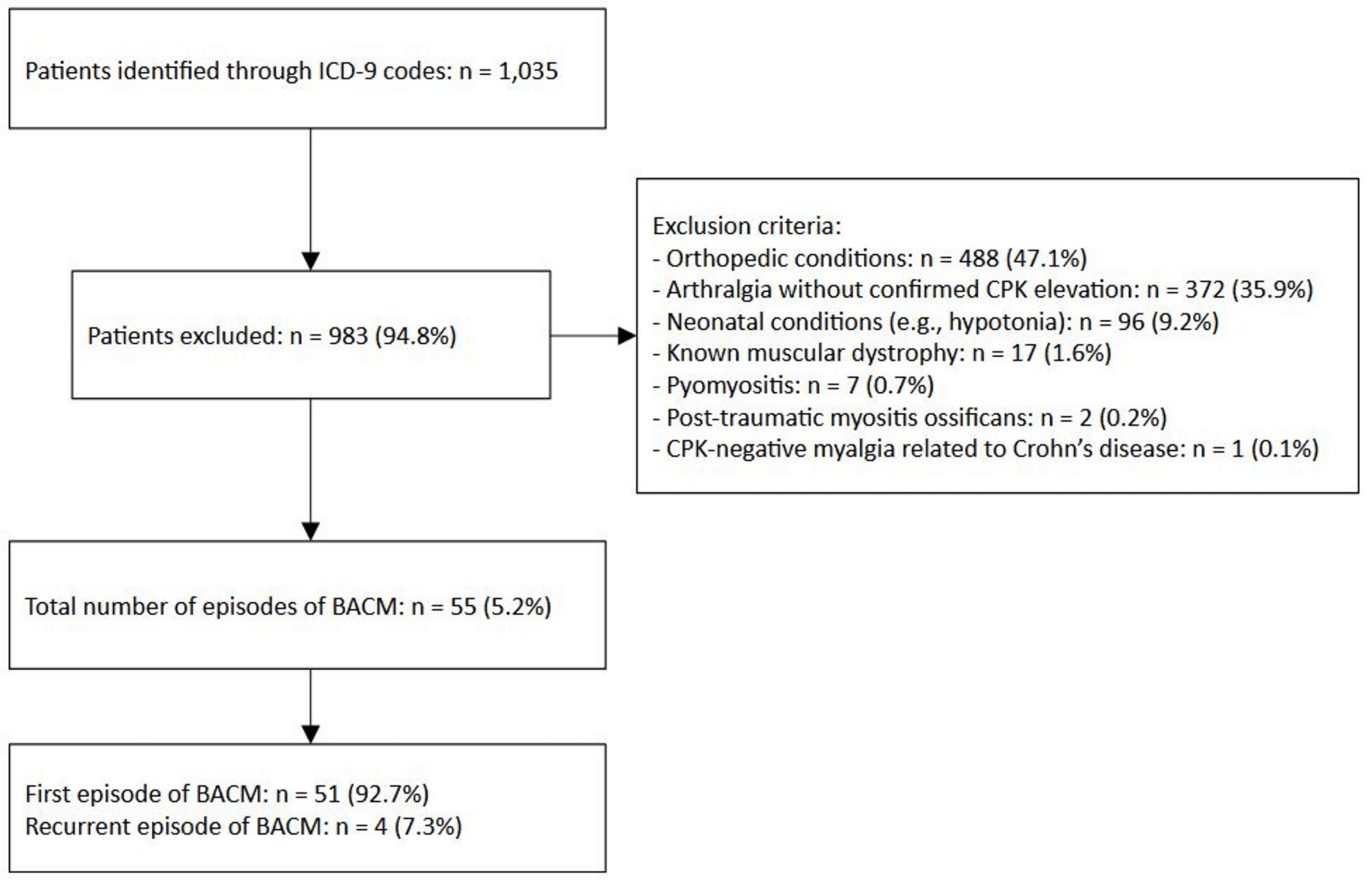
Sample size selection This flow chart shows the sample size selection performed to assess the final population of this multicenter retrospective study. ICD-9: International Classification of Diseases, 9th Revision; BACM: benign acute childhood myositis; CPK: creatine phosphokinase

A total of 1,035 patients were selected based on the previously mentioned codes. Of these, 488 (47.1%) were excluded due to orthopedic conditions; 372 (35.9%) were excluded due to arthralgia without confirmed CPK elevation; 96 (9.2%) due to neonatal conditions (e.g., neonatal hypotonia); 17 (1.6%) patients had a known muscular dystrophy; seven (0.7%) were excluded due to pyomyositis; two patients (0.2%) due to post-traumatic myositis ossificans; and one patient (0.1%) due to CPK-negative myalgia related to Crohn’s disease. A total of 51 (5.2%) patients, accounting for 55 episodes of BACM, were included in the study (Figure 1).

Data collection and measurements

Demographic, anamnesis, and clinical data were retrospectively collected from medical records. For all patients, when available, laboratory tests were collected, including CPK (baseline, minimum, peak), creatine kinase-MB isoenzyme (CK-MB), myoglobin, CRP, LDH, complete blood count with differential, renal function, liver cytolysis enzymes, and urine dipstick. The results of electrocardiographic investigations were described among the instrumental exams. Microbiological tests were collected and described when performed. Outcomes were described in terms of hospitalization and normalization of cytolysis indices, as well as any post-discharge assessments and follow-up. Patients were then divided into two groups, depending on whether the onset of myositis occurred before or after the pandemic period. Data from the two groups were compared to study potential differences, considering the variables already outlined.

Statistical analyses

The collected variables were analyzed as either continuous variables or proportions. Quantitative data were summarized using mean and standard deviation, while categorical data were presented as frequency (count) and relative frequency (percentage). Measured variables were analyzed using the Student's t-test, and categorical variables were analyzed using the Mann-Whitney U test or Fisher's exact test when appropriate. Unless otherwise indicated, two-tailed p values of < 0.05 were considered significant. Statistical analyses were performed using IBM SPSS Statistics for Windows, Version 21 (Released 2013; IBM Corp., Armonk, New York, United States).

Ethics approval

According to the policies of AULSS 7 Pedemontana, Institutional Review Board (IRB)/ethics committee approval was not required for this retrospective study involving completely anonymized patient data.

## Results

Population of the study

**Figure 2:**
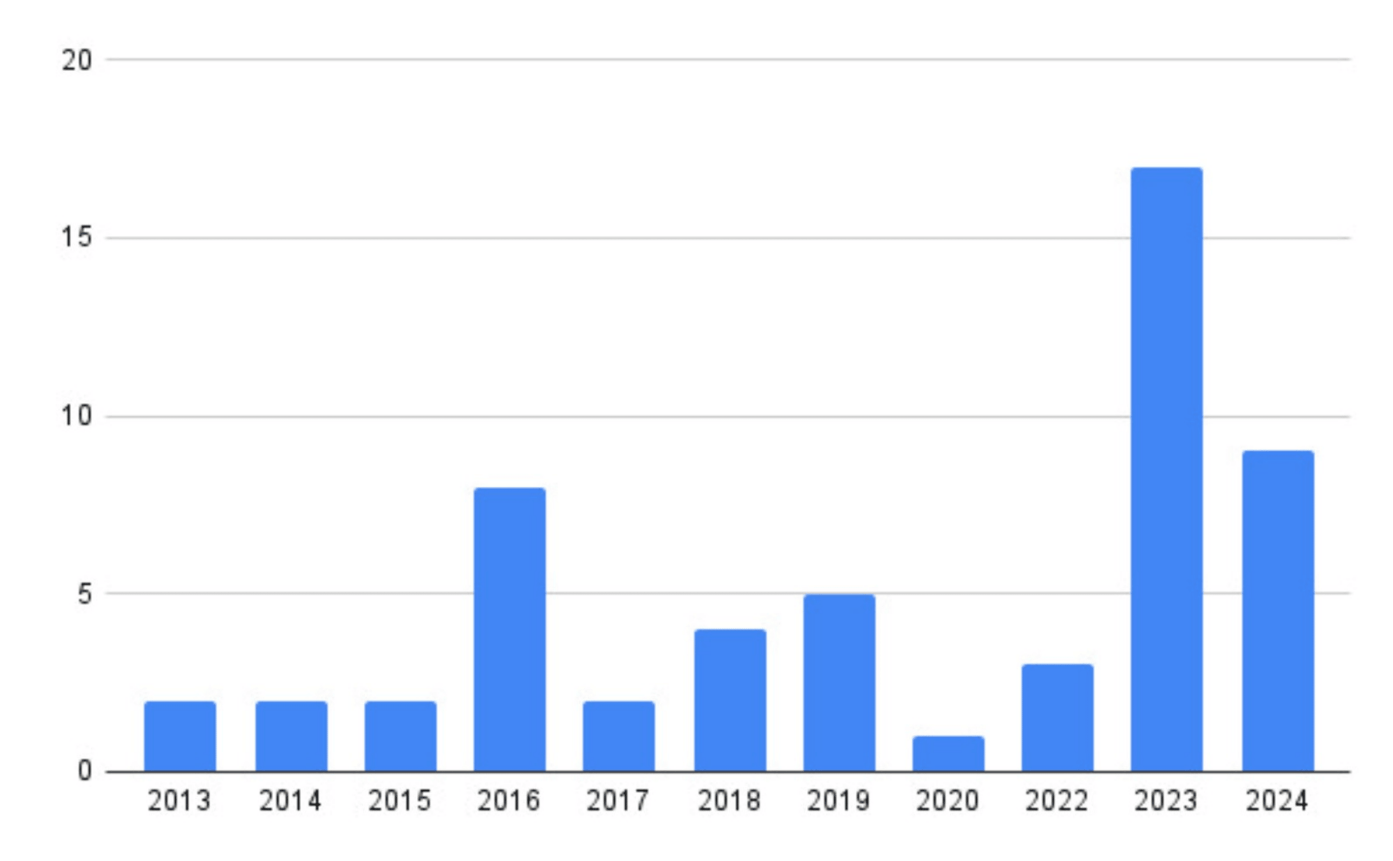
Incidence of benign acute childhood myositis This graph shows the incidence of benign acute childhood myositis from 2013 to 2024, highlighting a marked increase in myositis cases in the post-pandemic period.

A total of 51 patients with a clinical and laboratory diagnosis of myositis were included in the study, accounting for 55 different episodes of BACM, with an incidence of approximately 4.8 cases per 10,000 admissions to the ED of the involved hospitals. Approximately 35 cases, accounting for over 50% of the episodes, occurred between 2020 and 2024 (Figure 2).

**Figure 3:**
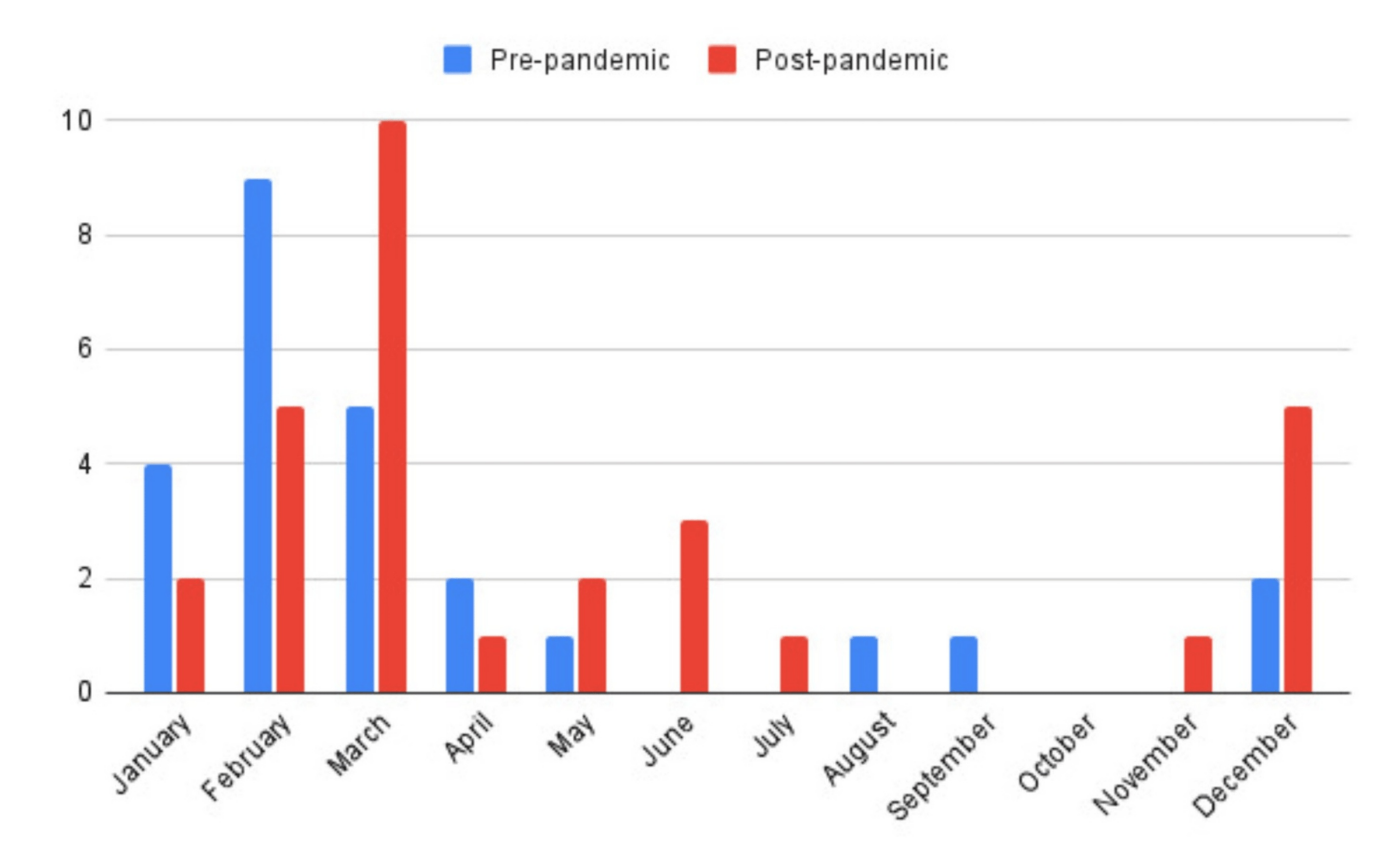
Monthly distribution of benign acute childhood myositis cases This graph highlights the seasonal trend of benign acute childhood myositis, showing a peak incidence during the winter months and only sporadic cases during the warmer seasons. No differences in seasonal distribution were observed between patients in the pre- and post-pandemic periods.

Most post-infectious myositis cases were observed during the winter months and early spring, with sporadic cases during summer (Figure 3).

The mean age of patients at admission was 8.1 years (± 3.6), with a clear predominance of male patients (64%). Four out of 51 patients (7.3%) experienced recurrent episodes of BACM. Only one patient (1.8%) in the observed population had a pre-existing condition (Tourette syndrome) not connected with BACM onset. All patients included in the study (100%) reported symptoms indicative of an intercurrent infection in the days preceding clinical evaluation, with an average time of approximately 5.8 days from the onset of infectious symptoms to the first presentation in the ED. Specifically, most patients exhibited fever (n = 51, 93%), respiratory symptoms including rhinitis (n = 18, 33%), pharyngitis (n = 19, 34%), asthenia (n = 18, 33%), and hyporexia (n = 13, 24%); a smaller proportion presented with gastrointestinal symptoms. Consistent with the current literature and understanding, the predominant symptom was lower limb pain (n = 49, 89%), followed by refusal of support (n = 30, 55%), often characterized by a wide-based gait (n = 17, 31%). The onset symptoms of BACM, which facilitate diagnosis when correlated with laboratory findings, are highlighted in Table 1.

**Table 1:** Clinical and demographic data of patients with benign acute childhood myositis The table summarizes the main clinical and demographic characteristics of patients with benign acute childhood myositis; values are expressed as number (percentage) or mean ± standard deviation. BACM: benign acute childhood myositis

Variable	Overall	Pre-pandemic	Post-pandemic	P-value
BACM episodes, n (%)	55 (100)	25 (45)	30 (55)	-
Gender (male), n (%)	35 (64)	16 (64)	19 (63)	0.95
Age, years	8.1 (± 3.6)	8.6 (± 3.7)	7.6 (± 3.5)	0.20
Days of previous symptoms	5.8 (± 3.5)	5.8 (± 3.8)	5.8 (± 3.3)	0.48
Cought, n (%)	25 (45)	12 (48)	13 (43)	0.73
Rhinitis, n (%)	18 (33)	7 (28)	11 (37)	0.50
Vomit, n (%)	8 (14)	4 (16)	4 (13)	0.78
Diarrhea, n (%)	8 (14)	4 (16)	4 (13)	0.78
Faringitis, n (%)	19 (34)	8 (32)	11 (37)	0.72
Asthenia, n (%)	18 (33)	6 (24)	12 (40)	0.21
Fever, n (%)	51 (93)	22 (88)	29 (97)	0.25
Hyporexia, n (%)	13 (24)	3 (12)	10 (33)	0.05
Upper limbs pain, n (%)	3 (6)	2 (8)	1 (3)	0.47
Lower limbs pain, n (%)	49 (89)	22 (88)	27 (91)	0.81
Widespread pain, n (%)	14 (25)	7 (28)	7 (23)	0.73
Refusal of support, n (%)	30 (55)	12 (48)	18 (60)	0.70
Wide base, n (%)	17 (31)	9 (36)	8 (27)	0.38
Toe walking, n (%)	14 (25)	7 (28)	7 (23)	0.46

Electrocardiogram and laboratory data

The average CPK levels on admission were 2982 IU/L, with a peak CPK level around 3620 IU/L. CPK peak appears to be set at seven days since the onset of the viral illness. Aspartate aminotransferase (AST) and alanine aminotransferase (ALT) levels were 170 IU/L and 51 IU/L, respectively, with peaks of 217 IU/L and 76 IU/L, reached by days 8 and 8.5, respectively, from infectious symptoms. Inflammatory markers were only slightly elevated (mean CRP 1.2 mg/dL). In five out of 55 episodes (9%), patients showed slight alterations in renal function indices. Among the four patients with recurrent BACM, three (75%) did not show significant differences concerning CPK values when compared with patients with a single episode of BACM. One patient with recurrence showed remarkably high values of CPK (with peak until 45000 IU/L - first episode - and 7938 IU/L - second episode) with renal impairment and rhabdomyolysis. Screening for neuromuscular diseases yielded negative results. Urinalysis revealed that nine (16%) patients had traces of hematuria, while eight (14%) had mild proteinuria. In 15 episodes (27%), patients underwent electrocardiographic analysis; none of these showed significant electrocardiogram (ECG) abnormalities. Laboratory findings can be found summarized in Table 2.

**Table 2:** Laboratory data of patients with benign acute childhood myositis The table summarizes the main laboratory characteristics of cases with benign acute childhood myositis; values are expressed as number (percentage) or mean ± standard deviation. BACM: benign acute childhood myositis; CRP: C-reactive protein; PLT: platelets; Hb: hemoglobin; CPK: creatine phosphokinase; LDH: lactate dehydrogenase; AST: aspartate aminotransferase; ALT: alanine aminotransferase

Variable	Overall	Pre-pandemic	Post-pandemic	P-value
BACM episodes, n (%)	55 (100)	25 (45)	30 (55)	-
CRP (mg/dL)	1.2 (± 3.1)	0.9 (± 2.5)	1.5 (± 3.5)	0.55
CRP peak (mg/dL)	1.5 (± 3.3)	1.2 (± 2.5)	1.9 (± 3.9)	0.23
White blood cells (/mmc)	6562 (± 3981)	5869 (± 3187)	7136 (± 4509)	0.23
Neutrophils (/mmc)	4197 (3800)	3691 (± 2985)	4598 (± 4351)	0.37
Lymphocytes (/mmc)	1854 (± 862)	1824 (± 1046)	1877 (± 701)	0.83
Eosinophils (/mmc)	52 (± 72)	63 (± 85)	44 (± 61)	0.36
Basophils (/mmc)	26 (± 22)	30 (± 25)	24 (± 19)	0.32
PLT (/mmc)	216000 (± 74000)	186000 (± 56500)	246600 (± 77000)	0.002
Hb (mg/dL)	13.4 (± 1.42)	13.5 (± 1.0)	13.3 (±1.7)	0.57
CPK (IU/L)	2982 (± 6297)	2161 (± 2704)	3639 (± 8104)	0.35
CPK peak (IU/L)	3620 (± 6472)	2385 (± 2783)	4567 (± 8191)	0.18
CPK peak (days)	1.5 (± 0.7)	1.5 (± 0.7)	1.5 (± 0.7)	1
CPK increase (IU/L)	597 (± 1562)	146 (± 605)	928 (± 1939)	0.03
CPK normalization (days)	6.7 (± 5.7)	4.2 (± 3.3)	8.5 (± 6.4)	0.004
LDH (IU/L)	427 (± 352)	428 (± 242)	427 (± 403)	0.99
Myoglobin (mcg/L)	1519 (± 2045)	447 (± 385)	1811 (± 2229)	0,07
Myoglobin peak (mcg/l)	1452 (± 1987)	458 (± 303)	1784 (± 2209)	0,06
AST (IU/L)	170 (± 465)	112 (± 85)	220 (± 629)	0.38
AST peak (IU/L)	217 (± 617)	122 (± 92)	295 (± 827)	0.28
ALT (IU/L)	51 (± 93)	44 (± 23)	56 (± 126)	0.63
ALT peak (IU/L)	76 (± 159)	57 (± 33)	93 (± 214)	0.38
Creatinin (mg/dL)	0.52 (± 0.22)	0.52 (± 0.18)	0.53 (± 0.24)	0.93
Urea (mg/dL)	24 (± 11)	24 (± 9)	25 (± 12)	0.75
Urine specific gravity	1017 (± 7)	1017 (± 8)	1017 (± 7)	0.89
Urine pH	6.1 (± 0.7)	6.3 (± 0.6)	5.9 (0.9)	0.09
Urine Hb, n (%)	9 (16)	3 (12)	4 (15)	0.51
Urine protein, n (%)	8 (14)	3 (12)	3 (11)	0.74

Approximately 28 (51%) patients underwent some form of microbiological investigation. Among these, 12 (23%) had serological tests, six (11%) had stool pathogen tests, and 21 (38%) had nasopharyngeal swabs for influenza A/B viruses or film-array tests for viral polymerase chain reaction (PCR). In addition, all of the patients since 2020 (n = 30, 55%) were tested for hCoV infection. In our population, the detected pathogens included 10 (42%) influenza B virus, six (25%) influenza A virus, three (12%) rotavirus, two (8%) adenovirus, one (4%) parainfluenza virus type 2, one (4%) EBV, and one (4%) *M. pneumoniae* (Table 3).

**Table 3:** Microbiological data of patients with benign acute childhood myositis The table summarizes the pathogens identified in patients with benign acute childhood myositis, along with their respective frequencies and the most common detection methods.

	N (%)	Pharyngeal swab	Blood serum	Stool swab
Influenza B virus	10 (42)	9	1	0
Influenza A virus	6 (25)	5	1	0
Rotavirus	3 (12)	0	0	3
Adenovirus	2 (8)	1	0	1
Parainfluenza virus type 2	1 (4)	1	0	0
Epstein-Barr virus	1 (4)	0	1	0
Mycoplasma pneumoniae	1 (4)	0	1	0

About 27 (49%) patients were not subjected to any etiological investigation. Among those tested, microorganisms were identified in 20 (71%) patients.

Management and outcomes

Approximately 11 (20%) cases were discharged directly from the ED to home, with recommendations for longitudinal follow-up by their primary care pediatrician. About 11 (20%) patients were discharged after a brief observation period (less than 36 hours), while 33 (60%) patients were admitted to pediatric wards. Intravenous therapy in hospitalized patients lasted an average of 2.7 days, with an average hospital stay of 3.2 days (Table 4).

**Table 4:** Management of patients with benign acute childhood myositis The table summarizes the discharge modalities and management of patients with benign acute childhood myositis, comparing the pre-pandemic and post-pandemic periods. Values are expressed as numbers (percentages) or mean ± standard deviation. BACM: benign acute childhood myositis; ED: emergency department

	Overall	Pre-pandemic	Post-pandemic	P-value
BACM episodes, n (%)	55 (100)	25 (45)	30 (55)	-
ED discharge, n (%)	11 (20)	6 (24)	5 (17)	0.51
Brief ED observation, n (%)	11 (20)	7 (28)	4 (13)	0.18
Hospitalization, n (%)	33 (60)	12 (48)	21 (70)	0.10
Intravenous hydration, n (%)	41 (74)	16 (64)	25 (83)	0.11
Treatment duration, days	2.7 (± 2.3)	2.3 (± 2.2)	3.1 (2.4)	0.13
Hospital stay, days	3.2 (± 2.4)	2.9 (± 2.4)	3.4 (± 2.6)	0.24

Rehydrating intravenous therapy was performed by administering fluids at a volume equal to 100-140% of the maintenance calculated according to the Holliday-Sager formula. None of the patients experienced a recurrence of symptoms after being discharged home. Only one patient (1.8%) underwent further evaluation for potential primary neuromuscular disease, which subsequently yielded negative results.

Pre-pandemic (2012-2019) versus post-pandemic (2020-2024) periods

An increase in BACM cases has been observed over the years, with an incidence of 6.5/10,000 pediatric ED admissions between 2020 and 2024, while the pre-COVID-19 period had an incidence of 3,6/10,000 admissions. Specifically, approximately 30 cases of BACM (55%) occurred during the post-pandemic period following SARS-CoV-2. The patients were divided into two groups according to the period of the occurrence of symptoms, sorting them into group one (pre-pandemic BACM, n = 25, 45%) and group two (post-pandemic BACM, n = 30, 55%). When comparing the two groups, no significant differences were noted regarding the distribution of BACM by sex, age, or etiology. There was a higher frequency of patients with hyporexia in the second group (n = 10, 33% vs. n = 3, 12%, p = 0.05), while no other significant differences were noted considering infectious symptoms. Notably, slightly higher levels of CPK, LDH, myoglobin, AST, and ALT were observed in patients with BACM onset post-SARS-CoV-2, even though the differences did not reach statistical significance. A statistically significant difference was observed in CPK elevation following the first hospital admission between the two patient groups, with an increase of 146 IU/L and 928 IU/L in the pre- and post-pandemic periods, respectively (p = 0.03). No differences were found regarding renal function (creatinine and urea); nevertheless, a higher prevalence of proteinuria or presence of hemoglobin at the urinalysis was noted in the second group of patients. Instead, these patients more frequently presented with refusal of support as previously shown in Table 1. Consistent with the elevated CPK levels, there was an increased trend toward hospitalization in the post-COVID period (n = 12, 48% vs. n = 21, 70%, p = 0.10), with longer treatment durations and hospital stays in terms of absolute values (2.36 vs. 3.07 days and 2.92 vs. 3.40 days, respectively), with no statistical significance when performing Fisher’s exact test (p = 0.13 and 0.24 respectively). Regarding CPK normalization, the lowest CPK values observed in the two patient cohorts (p = 0.09) occurred at 4.2 days and 8.5 days after the first ED visit in the pre-pandemic and post-pandemic groups, respectively (p = 0.004). These data were previously summarized in Table 4.

## Discussion

BACM is a condition of presumably multifactorial origin, generally presenting with a benign course [8]. Consistent with existing studies, we observed that most BACM cases occur in predominantly male patients, with a mean age of approximately eight years. This characteristic distribution by sex and age suggests an individual predisposition to developing muscle inflammation following an infectious stimulus, although the pathophysiology of this condition remains incompletely understood.

The influenza virus, particularly type B, is recognized as the leading cause of BACM [9]. However, our study confirms that other viral pathogens and atypical bacteria, such as *M. pneumoniae*, may also play a role [10]. Although SARS-CoV-2 has been described as a potential causative agent of BACM [3], none of the patients in our cohort tested positive for this pathogen, despite all being screened upon admission. Nevertheless, a seeming increase in BACM cases has been noted in recent years, particularly in the post-pandemic period, as previously reported by Attaianese et al. in a recent 2023 retrospective study [11]. There seems to be a similar trend when comparing influenza virus occurrence in pre-pandemic and post-pandemic periods [12-14]. The increase in influenza cases likely does not fully account for the higher incidence of BACM, which is known to affect only a small minority of patients with influenza A/B infection. In recent years, and particularly in the post-COVID-19 period, several diseases have shown an increased incidence, often with greater virulence than in the past. This is the case, for example, with infections caused by other pathogens such as enteroviruses or parvovirus B19, which have been associated with a concurrent rise in cases of encephalitis and myocarditis. To explain these findings, several authors have proposed the existence of an "immunity gap" in the new generation of children and adolescents, particularly in pediatric patients who experienced significantly reduced exposure to common pathogens during the quarantine and isolation period [15-17]. Moreover, in some countries, there has been a decline in adherence to influenza vaccination campaigns, which could contribute to increased circulation and pathogenic potential of the virus [18].

In our cohort, we compared BACM cases identified during the pre-SARS-CoV-2 period with those diagnosed in the post-pandemic era. We found that CPK levels seemed to be higher in post-pandemic BACM cases than in pre-pandemic cases, while the increase in CPK following admission to the ED was significantly greater in post-pandemic BACM patients. Similarly, increased levels of other muscle cytolysis markers, such as LDH, myoglobin, AST, and ALT, were observed (even if with no statistical significance). These differences in laboratory values, both at presentation and at peak levels, might correspond to variations in clinical symptoms. Patients in the post-pandemic group showed higher percentages of refusal to ambulate, whereas those in the pre-pandemic group more frequently retained the ability to walk, albeit with greater difficulty and compensatory mechanisms (e.g., tiptoe or wide-based gait).

This apparent increase in the severity of symptoms and laboratory findings in post-pandemic patients inevitably influenced their clinical management. Currently, no officially recognized guidelines exist for the management of BACM in pediatric EDs. Some authors, such as Brisca et al., have proposed management protocols suggesting hospitalization for patients with CPK levels exceeding 1500 IU/L at admission, renal impairment, or significant red flags (e.g., recurrent episodes, age <2 years, or a family history of neuromuscular disorders) [2]. Others suggest performing screening tests for muscular and metabolic disorders in recurrent myositis and/or cases with CPK elevation above 5000 IU/L [6,11]. Conversely, other authors, such as Costa Azevedo et al., emphasize the benign and self-limiting nature of BACM, arguing that it often leads to unnecessary hospitalizations and can be managed conservatively at home [1,19,20]. In our cohort, consistent with other case series in the literature, approximately 22 (40%) patients were discharged home directly from the ED or after a brief observation period. However, 33 (60%) patients were hospitalized for intravenous hydration. The number of hospitalizations apparently increased in the post-pandemic period, with correspondingly longer hospital stays (albeit with no statistical significance).

The independent variables significantly associated with hospitalization in our cohort included CPK levels on admission (p = 0.05) and at peak (p = 0.02), age at presentation (with higher hospitalization rates in older patients, p = 0.04), renal impairment (p = 0.05), and elevated blood urea levels (p < 0.0001). Interestingly, the presence of vomit or diarrhea seems to be inversely correlated with the need for hospitalization in patients with increased CPK levels (respectively, p = 0.001 and p = 0.001). Based on our findings, hospitalization appears indicated in cases of elevated creatinine or dark urine output, urea levels above the 75th percentile of normal, CPK levels exceeding 2500 IU/L on admission with refusal to deambulate, or inability to maintain adequate oral hydration.

Concerning patients with borderline indications, we observed that post-pandemic cases showed an average increase in CPK levels of over 900 IU/L following the first hospital admission (± 1900 IU/L). We therefore recommend observing patients for at least 24-36 hours if CPK levels are between 1000 IU/L and 2500 IU/L, particularly with recent-onset symptoms, to provide intravenous hyperhydration and repeat laboratory tests after 24 hours. The CPK peak often occurs approximately 1.5 days after the first hospital admission, with a median of 7.5 days of flu-like symptoms preceding medical evaluation. Therefore, we suggest performing blood tests in all patients, regardless of discharge or hospitalization status, around days 7-9 following symptom onset, and follow-up testing 7-10 days post-discharge to ensure normalization. As various authors previously reported, we were dealing with several patients with values of CPK far above 5000 IU/L with no recurrence and a complete resolution over the following years. We suggest performing analyses for muscular and metabolic disorders only when treating patients with CPK values above 10000 IU/L, with recurrence without microbiological identification, or with atypical presentation. Patients with CPK values above 10000 IU/L should be strictly monitored for an early assessment of eventual renal failure.

Study limitations

This study has several limitations that should be acknowledged. First, its retrospective design inherently limits the ability to control for confounding variables and is subject to selection and reporting biases. Data were collected from medical records, which may vary in completeness and accuracy across institutions and individual cases. Second, although the study was conducted across three centers, the relatively small sample size of 55 episodes limits the statistical power of our findings. As a result, the ability to perform subgroup analyses or draw robust conclusions from statistical associations is reduced, and the generalizability of the results may be limited. Third, a considerable proportion of patients did not undergo microbiological testing. This limits our ability to assess the role of specific pathogens, particularly viruses, in the development of BACM, and it may have led to underreporting of potential etiological factors. Fourth, in the absence of standardized national or international guidelines for the management of BACM, clinical decision-making was highly heterogeneous. Diagnostic workups and therapeutic approaches varied between institutions and physicians, reflecting individual clinical judgment rather than protocol-driven care. This heterogeneity may have introduced variability in the data, potentially affecting outcome comparisons. Fifth, there was likely variability in the clinical criteria used to diagnose BACM across centers, especially in milder cases, given the lack of universally accepted diagnostic standards. This could have led to inconsistencies in case inclusion and differences in the threshold for hospitalization or laboratory testing. Finally, systematic long-term follow-up was not available for most patients, limiting our ability to evaluate outcomes beyond the acute phase. In particular, we cannot exclude the possibility of recurrent episodes or delayed complications in some cases.

Despite these limitations, the study provides valuable multicenter data on the changing epidemiology and clinical patterns of BACM in the pre- and post-COVID-19 era, highlighting the need for greater diagnostic standardization and prospective investigation.

## Conclusions

BACM is a benign, post-infectious, and potentially recurrent condition that typically resolves spontaneously within approximately 15 days of onset. Its pathophysiology remains incompletely elucidated. The apparent increase in cases and in severity in recent years highlights the need to establish clear guidelines for its management, to prevent both excessive invasive testing and the underestimation of potentially severe aspects such as renal impairment.

All patients with compatible symptoms should undergo blood tests, including CPK, AST, ALT, CPK-MB, LDH, myoglobin, complete blood count, creatinine, urea, and uric acid, along with a urine dipstick, ECG, and, if possible, influenza A/B swab testing. Patients with mild symptoms, low CPK levels, no renal impairment, and good family compliance may be discharged home with parental reassurance about the benign nature of the condition.

## References

[1] Majava E, Renko M, Kuitunen I (2024). Benign acute childhood myositis: a scoping review of clinical presentation and viral etiology. Eur J Pediatr.

[2] Brisca G, Mariani M, Pirlo D (2021). Management and outcome of benign acute childhood myositis in pediatric emergency department. Ital J Pediatr.

[3] Tekin E, Akoğlu HA (2022). From influenza to SARS-CoV-2: etiological evaluation of acute benign childhood myositis. Acta Neurol Belg.

[4] Jiang S, Li J, Cao J, Ou Y, Duan Y, Gan X (2024). Clinical characteristics of 118 pediatric patients with acute benign myositis associated with influenza A virus infection. Pediatr Infect Dis J.

[5] Öztürk B, Göktuğ A, Bodur İ (2022). Benign acute childhood myositis: factors associated with muscle symptoms and resolution. Pediatr Int.

[6] Turan C, Yurtseven A, Cicek C, Keskin G, Saz EU (2022). Benign acute childhood myositis associated with influenza A/B in the paediatric emergency department and the efficacy of early-onset oseltamivir. J Paediatr Child Health.

[7] Santos JA, Albuquerque C, Lito D, Cunha F (2014). Benign acute childhood myositis: an alarming condition with an excellent prognosis!. Am J Emerg Med.

[8] Rosenberg T, Heitner S, Scolnik D, Levin Ben-Adiva E, Rimon A, Glatstein M (2018). Outcome of benign acute childhood myositis: the experience of 2 large tertiary care pediatric hospitals. Pediatr Emerg Care.

[9] Pari P, Manikandan E, Jose P, Kumar PP, Fermendenz S, Dhodapkar R (2023). Benign acute childhood myositis following human Influenza B virus infection - a case series. Trop Doct.

[10] D'Amico S, Gangi G, Barbagallo M (2022). Benign acute childhood myositis: our experience on clinical evaluation. Neuropediatrics.

[11] Attaianese F, Costantino A, Benucci C, Lasagni D, Trapani S (2023). Benign acute children myositis: 5 years experience in a tertiary care pediatric hospital. Eur J Pediatr.

[12] Lin F, Liang JL, Guan ZX, Wu M, Yang LY (2024). Hospitalized children with influenza A before, during and after COVID-19 pandemic: a retrospective cohort study. BMC Pediatr.

[13] Chen HW, Chang MC, Wang TL, Shia BC, Wang SR (2025). Benign acute childhood myositis in the COVID-19 Era: how does it compare to influenza?. Eur J Pediatr.

[14] Lin F, Chen MT, Zhang L (2023). Resurgence of influenza A after SARS-CoV-2 omicron wave and comparative analysis of hospitalized children with COVID-19 and influenza A virus infection. Front Med (Lausanne).

[15] Cohen R, Ashman M, Taha MK (2021). Pediatric Infectious Disease Group (GPIP) position paper on the immune debt of the COVID-19 pandemic in childhood, how can we fill the immunity gap?. Infect Dis Now.

[16] Russcher A, van Boven M, Benincà E (2024). Changing epidemiology of parvovirus B19 in the Netherlands since 1990, including its re-emergence after the COVID-19 pandemic. Sci Rep.

[17] Poeta M, Moracas C, Calò Carducci FI (2024). Outbreak of paediatric myocarditis associated with parvovirus B19 infection in Italy, January to October 2024. Euro Surveill.

[18] Gerussi V, Peghin M, Palese A (2024). SARS-CoV-2 and influenza vaccine hesitancy during the COVID-19 pandemic in a dynamic perspective. Hum Vaccin Immunother.

[19] Costa Azevedo A, Costa E Silva A, Juliana Silva C, Poço Miranda S, Costa M, Martinho I (2022). Benign acute childhood myositis: a 5-year retrospective study. Arch Pediatr.

[20] Jarkoc T, Cengic A, Selmanovic V (2025). Characteristics of patients with benign acute childhood myositis (BACM). Mater Sociomed.

